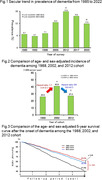# 37‐year secular trends in prevalence, incidence, and survival rate of dementia in a Japanese community: the Hisayama Study

**DOI:** 10.1002/alz.088678

**Published:** 2025-01-09

**Authors:** Tomoyuki Ohara, Toshifumi Minohara, Taro Nakazawa, Jun Hata, Mao Shibata, Toshiharu Ninomiya

**Affiliations:** ^1^ Graduate School of Medical Sciences, Kyushu University, Fukuoka Japan

## Abstract

**Background:**

Few population‐based prospective studies have investigated whether the prevalence, incidence, and survival rate of dementia in a community have changed since the 2010s.

**Method:**

Seven cross‐sectional surveys of dementia were conducted among residents of a Japanese community, aged ≥65 years, in 1985, 1992, 1998, 2005, 2012, 2017, and 2022. We also established three cohorts consisting of the residents of this age group without dementia in 1988 (n = 803), 2002 (n = 1,231), and 2012 (n = 1,521) and each was followed for 10 years. Trends in the prevalence of dementia were tested by using the logistic regression model. The age‐ and sex‐adjusted incidence and survival rate of dementia were compared between cohorts using the Cox proportional hazards model.

**Result:**

The crude prevalence of dementia increased with time from 1985 to 2012 (6.7% in 1985, 5.7% in 1992, 7.1% in 1998, 12.5% in 2005, and 17.9% in 2012, p for trend <0.01), while the crude prevalence of dementia decreased with time from 2012 to 2022 (17.9% in 2012, 15.6% in 2017, and 11.9% in 2022, p for trend <0.01). These secular changes did not change even after standardizing by age. The age‐ and sex‐adjusted incidence of dementia significantly increased from the 1988 cohort to the 2002 cohort (adjusted hazard ratio [aHR] = 1.7, 95% confidence intervals [CI] = 1.4‐2.1). However, the age‐ and sex‐adjusted incidence of dementia significantly decreased from the 2002 cohort to 2012 cohort (aHR = 0.6, 95% CI = 0.5‐0.7). The age‐ and sex‐adjusted 5‐year survival rate of dementia significantly improved from the 1988 cohort to the 2002 cohort (47.3% to 65.2%, p<0.01), while no secular change was observed from the 2002 cohort to the 2012 cohort (65.2% to 58.3%, p = 0.39).

**Conclusion:**

The decreasing trends in the prevalence and incidence of dementia were observed since 2012 in the Japanese older population. The decline in the incidence of dementia may be due to prevention of lifestyle‐related diseases, such as hypertension and diabetes, and improvement of proper management of the disease, as well as awareness and promotion of healthy lifestyle behaviors.